# Network design and analysis for multi-enzyme biocatalysis

**DOI:** 10.1186/s12859-017-1773-y

**Published:** 2017-08-10

**Authors:** Lisa Katharina Blaß, Christian Weyler, Elmar Heinzle

**Affiliations:** Biochemical Engineering Institute, Saarland University, Campus A1.5, Saarbrücken, 66123 Germany

**Keywords:** Network design, Network analysis, Pathway, Biocatalysis, Multi-enzyme catalysis, Mixed-integer linear program, Path-finding, Side reactions, Thermodynamics, Synthetic biology

## Abstract

**Background:**

As more and more biological reaction data become available, the full exploration of the enzymatic potential for the synthesis of valuable products opens up exciting new opportunities but is becoming increasingly complex. The manual design of multi-step biosynthesis routes involving enzymes from different organisms is very challenging. To harness the full enzymatic potential, we developed a computational tool for the directed design of biosynthetic production pathways for multi-step catalysis with in vitro enzyme cascades, cell hydrolysates and permeabilized cells.

**Results:**

We present a method which encompasses the reconstruction of a genome-scale pan-organism metabolic network, path-finding and the ranking of the resulting pathway candidates for proposing suitable synthesis pathways. The network is based on reaction and reaction pair data from the Kyoto Encyclopedia of Genes and Genomes (KEGG) and the thermodynamics calculator eQuilibrator. The pan-organism network is especially useful for finding the most suitable pathway to a target metabolite from a thermodynamic or economic standpoint. However, our method can be used with any network reconstruction, e.g. for a specific organism. We implemented a path-finding algorithm based on a mixed-integer linear program (MILP) which takes into account both topology and stoichiometry of the underlying network. Unlike other methods we do not specify a single starting metabolite, but our algorithm searches for pathways starting from arbitrary start metabolites to a target product of interest. Using a set of biochemical ranking criteria including pathway length, thermodynamics and other biological characteristics such as number of heterologous enzymes or cofactor requirement, it is possible to obtain well-designed meaningful pathway alternatives. In addition, a thermodynamic profile, the overall reactant balance and potential side reactions as well as an SBML file for visualization are generated for each pathway alternative.

**Conclusion:**

We present an in silico tool for the design of multi-enzyme biosynthetic production pathways starting from a pan-organism network. The method is highly customizable and each module can be adapted to the focus of the project at hand. This method is directly applicable for (i) in vitro enzyme cascades, (ii) cell hydrolysates and (iii) permeabilized cells.

**Electronic supplementary material:**

The online version of this article (doi:10.1186/s12859-017-1773-y) contains supplementary material, which is available to authorized users.

## Background

While thousands of enzymes are already known, numerous new enzymes or new enzymatic activities are still discovered every year. Many of these biocatalysts accept multiple substrates and even catalyze different reactions. From a biotechnological point of view, the enzymatic potential of nature can be considered an extremely versatile tool potentially giving access to countless valuable products ranging from bulk chemicals to most complex drug compounds. The methods for such syntheses can range from using single isolated enzymes over multi-enzyme systems or enzyme cascades up to syntheses with cell lysates or permeabilized cells [1].

However, the full exploration of the enzymatic potential is often hampered by the sheer amount and complexity of available reaction data. When manually designing a multi-step synthesis route to a certain metabolic intermediate, the network of alternative synthesis pathways quickly grows highly complex as more reaction steps are introduced. Additionally, assembling all reactions that lead to each reactant is extremely time consuming. The manual determination of the most suitable pathway candidate is challenging as multiple aspects such as thermodynamics, cofactor use, etc. need to be considered. To more easily harness the full potential of the enzymatic toolbox we developed a computational tool for the directed design of biosynthetic production pathways for interesting products in cell extracts and permeabilized cells.

The search for pathways in genome-scale metabolic networks is a common task of wide interest and there is a large variety of path-finding and pathway design methods. Most of those methods can be categorized into one of two types, namely stoichiometric methods and graph-based methods. Stoichiometric methods make use of the stoichiometry of a network to analyze the metabolism under the assumption of a steady-state condition. Popular and mathematically well understood methods are for example elementary flux modes [2] or flux balance analysis [3, 4].

Graph-based methods in general neglect stoichiometry and treat the networks as graphs in a mathematical sense and search for pathways based on connectivity [5], with the use of atom or atom group tracking [6–8], retrosynthesis [9, 10], heuristic search algorithms [11] or evolutionary algorithms [12]. In the last years, methods combining stoichiometry and structural properties of networks emerged, e.g. the so called carbon flux paths proposed by Pey et al. [13, 14].

However, the majority of these methods tackles the problem of finding pathways between two given metabolites and does not take into account a search starting with an arbitrary metabolite in the network. Another drawback of these methods for our focus of application is that most of them assume a steady-state condition for the major part of the network. This is valid for living cells or cells with intact membranes. In these cases the actual reactions are running in a cellular compartment that keeps all intermediates separated from the bioreactor, whereas in the case of enzyme cocktails and permeabilized cells the reaction compartment is identical to the bioreactor used. Examples of the latter type of reaction systems are becoming increasingly popular [15–23].

We thus propose a tool which encompasses the reconstruction of a genome-scale pan-organism metabolic network, the implementation of a path-finding algorithm and the ranking of pathway candidates for proposing suitable synthesis pathways starting from arbitrary substrates.

## Methods

**Fig. 1:**
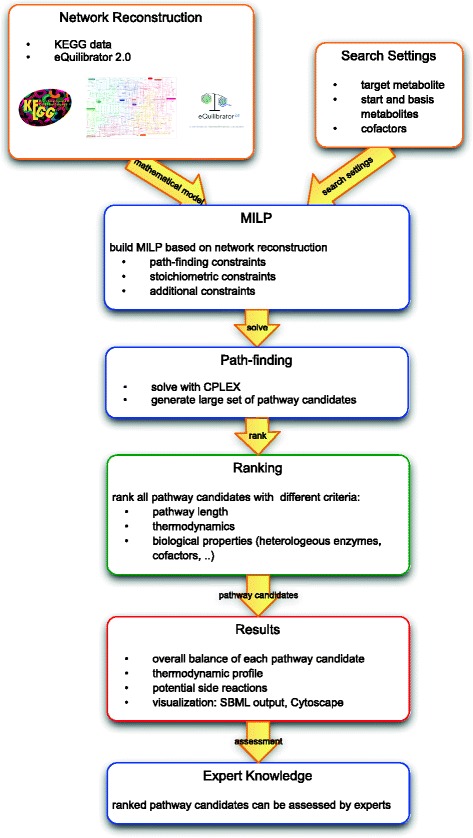
Workflow through the components of our tool. We start with a network reconstruction which is then used for path-finding with the presented MILP. The resulting pathway candidates are ranked according to the different ranking criteria

In the following we will present the individual parts of our method. Figure 1 shows the workflow through its different components.

The first step is the network reconstruction where the network is built with data from KEGG [24, 25] and the biochemical thermodynamics calculator eQuilibrator 2.0 [26, 27]. Details on how the network is compiled are given in section Network reconstruction. The path-finding in the network is based on an optimization algorithm developed by Pey et al. [13]. It combines graph-based path-finding and reaction stoichiometry in a mixed-integer linear program (MILP). The algorithm with our extensions is presented in detail in section Mathematical model. In a further stage the resulting pathway candidates are ranked using different criteria. We will give details on the ranking in section Filtering and ranking. The output is a list of ranked pathway candidates which can be assessed with expert knowledge to help determining the most suitable synthesis pathway for a desired product.

### Network reconstruction

We combine data from different KEGG databases and eQuilibrator 2.0 for the reconstruction of a pan-organism network with data from all organisms contained in KEGG release 78.1 from May 1, 2016.

#### Reaction and reaction pair data

The reaction network was reconstructed with COBRA Toolbox [28] using reactions from KEGG REACTION. We excluded reactions with the comments ’generic’ and ’incomplete’ in their data entries; reactions with ambiguous stoichiometry with stoichiometric coefficient n in the reaction equation; as well as reactions involving glycans with G numbers in KEGG.

From all remaining reactions in the model we built a network of reaction pairs, the so called arcs. A reaction pair is a biologically meaningful substrate-product pair in a reaction. We derived the arcs from the KEGG RPAIR database^1^ containing reaction pairs for each reaction. The reaction pairs in KEGG are classified into five categories [29] from which we used the main-pairs, describing the main changes on the substrates in a reaction and the trans-pairs which describe transferase reactions. We did not use the remaining three types cofac-pairs, ligase-pairs and leave-pairs. However, they can be included at user’s discretion.

Our network reconstruction comprises a total of 9038 reactions (10160 including reversible reactions), 7405 metabolites and 14803 arcs.

#### Thermodynamic data

The KEGG REACTION database does not contain any detailed information about reaction directions, so we incorporated thermodynamic data from the biochemical thermodynamics calculator eQuilibrator 2.0. The component contribution method used [27] provides different types of the reaction Gibbs energy. *Δ*_*r*_ *G*_′_° expresses the change of the Gibbs free energy of a reaction at a given pH and ionic strength *I* in 1 M concentration of the reactants. However, for metabolic reactions in cells it makes more sense to use physiologically meaningful concentrations. For *Δ*_*r*_ *G* ^′*m*^ the concentration of the reactants is thus set to 1 mM. For all calculations standard parameters are used which are a temperature of 25 °C (298.15 Kelvin), a pH of 7 and a pressure of 1 bar. We set the threshold for the discrimination of reversible and irreversibleto *Δ*_*r*_ *G* = 15 kJ/mol. Reactions without available thermodynamic data are considered irreversible in the direction given in the reaction equation from KEGG.

#### Network details

**Fig. 2:**
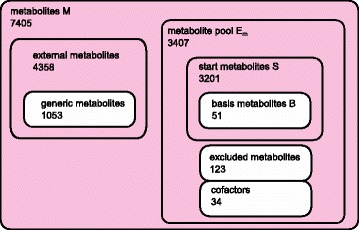
Venn diagram with the different metabolite categories in the network reconstruction. Metabolites M: all metabolites in the network; metabolite pool *E*_*m*_: metabolites considered available from start; start metabolites: all metabolites in the model contained in arcs with a molecular mass between 0 and 300; basis metabolites: expert-curated subset of start metabolites; cofactors: cofactors for enzymes; excluded metabolites: treated as cofactors; external metabolites: not contained in the metabolite pool, cannot be externally supplied; generic metabolites: marked as ’generic’ in their KEGG entry; the *light red* background indicates the set that can contain the product P

We categorize the metabolites in the model into different sets which we treat differently in our path-finding method. All sets are given in the Additional file 1. A Venn diagram of these sets is depicted in Fig. 2.

As *start metabolites* *S* we denote all metabolites that can be potential start points of a metabolite path. A metabolite path is a sequence of metabolites through the network connected by arcs. We compiled the list of possible start metabolites with all metabolites in the model contained in arcs with a molecular mass between 0 and 300. A subset of the start metabolites are the so called *basis metabolites* B. They are an expert-curated set of metabolites that are hubs of the arc network, easily available and inexpensive, such as D-glucose (C00031^2^) or pyruvate (C00022).

As *cofactors* we denote metabolites that are required for the activity of the enzymes catalyzing the reactions in the network but are not directly part of the reaction chain. We exclude arcs containing cofactors from the set of arcs to prevent biologically meaningless shortcuts in the network. The list is expert-curated and contains mono-, di- and triphosphates (e.g. AMP (C00020), ADP (C00008) and ATP (C00002)), electron carriers such as NAD ^+^ (C00003) and others. The mono- and diphosphates are usually not considered cofactors, but we chose to incorporate them into the list to avoid unnecessary interconversions between them on the pathway candidates.

The set of *excluded metabolites* is treated in the same way as the cofactors. It contains metabolites that are considered as freely available, such as water, oxygen or CO_2_.

As the *metabolite pool* *E*_*m*_ we denote the superset of metabolites we consider as freely available. This set consists of start metabolites, basis metabolites, cofactors and excluded metabolites.

As *external metabolites* we denote all metabolites that are not contained in the metabolite pool. They have to be produced in a production pathway and cannot be externally supplied. *Generic metabolites* are metabolites that are marked as ’generic’ in their KEGG entry, such as peptide (C00012) or protein (C00017). In our network we treat them as external metabolites and exclude arcs containing those metabolites from the arc network. The pool of external metabolites also contains metabolites with arcs that are not start metabolites as well as all other metabolites that are not part of any other set.

### Path-finding

In the following we introduce our method for finding pathway candidates in the network by means of a MILP.

#### Mathematical model

Given a metabolic model with the set of reactions *R* and the set of metabolites *M* we build the network of arcs. We also use the |*M*|-by- |*R*| stoichiometric matrix of the network, where each row corresponds to a metabolite and each column corresponds to a reaction. An entry in the matrix represents the stoichiometric value of a metabolite in the respective reaction, where negative values indicate a reactant and positive values indicate a product. Reversible reactions appear in the model as two different reactions with opposite directions.

#### MILP

The algorithm presented is based on an algorithm proposed by Pey et al. [13]. However, in comparison to the original algorithm we changed the problem statement. Pey et al. dealt with the question of finding the *K*-shortest flux paths between a given source and a target metabolite. Different from this problem statement we do not specify any specific starting metabolite, but our algorithm identifies suitable starting metabolites for finding a pathway to a target metabolite *P*.

In our definition, a *pathway* consists of two parts. The first part is a sequence of metabolites connected by reactions. It starts with a reaction that has one of the possible start metabolites as substrate and ends with a reaction with the desired target metabolite as a product. This part is called the *linear path*. The second part is a minimal set of reactions supplying substrates that are needed by the reactions on the path which are not contained in the metabolite pool. These are called *supplying reactions*.

We introduce the set of binary variables *u*_*ij*_ which are 1, if an arc from *i* to *j* is part of the linear path, and 0 otherwise (for *i,j*=1,…,|*M*|). The first constraint given by Eq. (1) establishes that there is exactly one arc on the linear path ending in the target metabolite *P*, whereas the second constraint in Eq. (2) assures that no arc on the linear path starts with *P*. The two constraints ensure that the target *P* is always the last node on each identified path and thus the path actually ends with the desired product. Both constraints have been adopted from [13].

1 $$ \sum_{i=1}^{|M|}u_{iP} = 1  $$

2 $$ \sum_{j=1}^{|M|}u_{P j} = 0  $$

Inequality (3) states that the number of arcs entering a node *l* from the set of possible start nodes *S* on the path is smaller or equal to the number of arcs leaving it.

3 $$ \sum\limits_{i=1}^{|M|} u_{il} \leq \sum\limits_{j=1}^{|M|} u_{lj} \quad l \in S; \quad l \neq P  $$

This means that a metabolite *l* is either the starting metabolite of a path ($\sum u_{il} = 0$ and $\sum u_{lj} = 1$) or the metabolite is an intermediate ($\sum u_{il} = \sum u_{lj}$). In the trivial case where *l* is not on the path, both sums are zero. The idea of the constraint has been adopted from [13]. However, we changed it to incorporate the set of starting metabolites, which has not been introduced in the original MILP.

For the set of basis metabolites *B* we introduce a constraint formulated in equation (4) stating that the number of arcs entering a node *l* from the set of basis metabolites *B* should be zero. This means that a basis metabolite can only appear as the first metabolite in a metabolite path and not as an intermediate.

4 $$ \sum\limits_{i=1}^{|M|} u_{il} = 0 \quad l \in B; \quad l \neq P  $$

For all other nodes *k* in the network except the target node *P* the number of in-going arcs must be equal to the number of out-going arcs, as given in constraint (5).

5 $$ \sum\limits_{i=1}^{|M|} u_{ik} = \sum\limits_{j=1}^{|M|} u_{kj} \quad k \in M \setminus S; \quad k \neq P  $$

This means that if an arc is entering an intermediate node *k*, then there must also be an arc leaving this node. Constraints (3) to (5) ensure that a path can only start with a start metabolite contained in the set of possible start nodes *S*. This constraint was taken from [13], but has been adapted for start metabolites.

Constraint (6), which was adopted from [13], forces nodes on a path to be unique, i.e. at most one arc can enter any given node.

6 $$ \sum\limits_{i=1}^{|M|} u_{ik} \leq 1, \quad k = 1,\dots, |M|  $$

Constraints (1) to (6) ensure that a solution contains a connected simple path from a start node of the set of start nodes *S* to a given end node *P*.

The next set of constraints deals with the feasibility of the linear path in the given network. Given are the stoichiometric coefficients *S*_*mr*_ for a metabolite *m* in reaction *r* (for *m*=1,…,|*M*|, *r*=1,…,|*R*|). The variables *v*_*r*_ assign each reaction *r* a non-negative flux. Constraint (7) expresses that the external metabolites are not necessarily balanced and can only be produced, but not be taken up. Only metabolites from the metabolite pool *E*_*m*_ containing the set of start metabolites, basis metabolites, cofactors and excluded metabolites can be taken up. This means that all substrates on the pathway must be producible with metabolites contained in the metabolite pool. This constraint was adopted from [13].

7 $$ \sum\limits_{r=1}^{|R|} S_{mr}v_{r} \geq 0, \quad \forall m \in E, m \notin E_{m}  $$

We added constraint (8) to make sure the target metabolite *P* can only be produced.

8 $$ \sum\limits_{r=1}^{|R|} S_{P r}v_{r} \geq 1,  $$

With constraints (9) and (10), (adopted from [13]), we introduce the binary variable *z*_*r*_ which is 1, when reaction *r* has a flux and 0 otherwise. All fluxes are scaled between 1 and a chosen positive value *Max* with *Max*≥1. This constraint relates fluxes in the flux distribution defined by *v*_*r*_ to reactions.

9 $$\begin{array}{*{20}l} z_{r} &\leq v_{r}, &r &= 1, \dots, R \end{array} $$

10 $$\begin{array}{*{20}l} \text{and} \quad v_{r} &\leq Max \cdot z_{r}, &r &= 1, \dots,R  \end{array} $$

Constraint (11) states that a reaction and its reverse cannot appear together in a valid flux distribution to exclude trivial cycles. This constraint was adopted from [13]).

11 $$\begin{array}{*{20}l} &z_{\lambda} + z_{\mu} \leq 1 \\ &\forall(\lambda,\mu) \in B = \left\{(\lambda, \mu)| \text{\(\lambda\) and \(\mu\) are reverse}\right\}  \end{array} $$

The path-finding and the stoichiometry constraints are linked through a linking constraint (12).

12 $$ \sum\limits_{r=1}^{|R|} d_{ijr} \cdot z_{r} \geq u_{ij} \quad i = 1, \dots,|M|; j = 1, \dots,|M|; i \neq j  $$

The binary coefficients *d*_*ijr*_ are 1, if there exists an arc between the metabolites *i* and *j* in reaction *r* and 0 otherwise. If an arc from *i* to *j* is used in the path (*u*_*ij*_=1) then at least one reaction *r* containing this arc (*d*_*ijr*_=1) has to be active. This constraint was adopted from [13]).

Constraints (7) to (12) define a valid flux distribution for the pathway ensuring that the found path is feasible.

The objective function of the problem is formulated in Eq. (13).

13 $$ Minimize \quad \sum\limits_{i=1}^{|M|} \sum\limits_{j=1, j \neq i}^{|M|} u_{ij} + \frac{1}{|R|+1}\sum\limits_{i=1}^{|R|} z_{i}  $$

As proposed by [13] we also minimize the number of arcs *u*_*ij*_ used but additionally we also minimize the number of active reactions on the whole pathway candidate. In contrast to [13] we are interested in finding pathways with different supplying reactions to provide different feasible pathway alternatives.

A solution to the MILP described by Eqs. (1) to (13) is a sequence of arcs given by the values of *u*_*ij*_ and the set of active reactions given by the values of *z*_*r*_. By minimizing the objective function we ensure that the linear path is connected and cycle-free and the number of active reactions and thus of supplying reactions is minimal. From the active reactions we determine those corresponding to the active arcs, denoted as *Z* ^′^. One solution represents one pathway candidate.

To find further solutions we have to exclude solutions with the same active arcs and the same reactions *Z* ^′^. Note that a valid new solution can have exactly the same set of active arcs as a previous solution if *Z* ^′^ is different, since an arc can be derived from more than one reaction. Let $U^{k}_{ij}$ be the value of *u*_*ij*_ for the *k*-th unique solution with respect to the metabolite path. To indicate that a solution is exactly the same as solution *k* regarding the metabolite path, we introduce a binary variable *s*_*k*_. When a solution is different from solution *k* regarding the metabolite path, *s*_*k*_ has to be 0 and 1 otherwise. Whenever we find a metabolite path $\phantom {\dot {i}\!}U^{k^{\prime }}$ we have not seen before, we introduce constraints (14), (15), (16) and a new binary variable $s_{k^{\prime }}$.

14 $$ \sum_{i}^{|M|} \sum_{j}^{|M|} U^{k'}_{ij} \cdot s_{k'} \leq \sum_{i}^{|M|} \sum_{j}^{|M|} U^{k'}_{ij} u_{ij}  $$

15 $$ \sum_{i}^{|M|} \sum_{j}^{|M|} \left(1-U^{k'}_{ij}\right)u_{ij} + s_{k'} |M|^{2} \leq |M|^{2}  $$

Constraints (14) and (15) establishes that, whenever we find a new solution *U* and $s_{k^{\prime }}$ is set to 1, we know that $U = U^{k^{\prime }}$. In more detail, constraint (14) ensures that if $s_{k^{\prime }}$ is 1 all arcs of solution *k* ^′^ are also active. Additionally, constraint (15) forbids *U* to contain any arc that was not present in $\phantom {\dot {i}\!}U^{k^{\prime }}$.

We denote the first metabolite in the path in solution *k* ^′^ by $\phantom {\dot {i}\!}\alpha ^{k^{\prime }}$.

16 $$ \sum_{i}^{|M|} \sum_{j}^{|M|}U^{k'}_{ij}u_{ij} - \sum_{i}^{|M|}u_{i\alpha^{k'}} - s_{k'} \leq \sum_{i}^{|M|} \sum_{j}^{|M|} U^{k'}_{ij} -1  $$

Constraint (16) ensures that a valid new solution has to fulfil one of the following three properties. It has either exactly the same metabolite path $\phantom {\dot {i}\!}U^{k^{\prime }}$; or at least one of the arcs from the previous metabolite path $\phantom {\dot {i}\!}U^{k^{\prime }}$ is not active; or all arcs from $\phantom {\dot {i}\!}U^{k^{\prime }}$ are active and one arc entering the first metabolite $\phantom {\dot {i}\!}\alpha ^{k^{\prime }}$ is active extending a previously found metabolite path. This constraint also ensures that $\phantom {\dot {i}\!}s_{k^{\prime }}$ is set to 1 if $\phantom {\dot {i}\!}U = U^{k^{\prime }}$.

Constraint (17) is always added for each new solution. Assume the found metabolite path is the same from solution *k* (*U* ^*k*^). Let $Z^{\prime {l}}_{i}$ indicate whether reaction *i* is active in solution *l* and corresponds to an active arc in *U* ^*k*^. The number of ones in *Z* ^′*l*^ is denoted by *m*_*l*_. This constraint prevents to find a second solution that is exactly the same as a previously found solution with regard to both linear path and reactions.

17 $$ \sum_{i}^{|R|} Z^{\prime{l}}_{i} z_{i} + s_{k} |R| \leq m_{l}-1 + |R|  $$

**Fig. 3:**
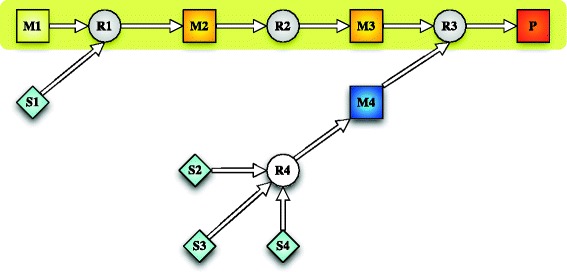
Exemplary pathway illustrating a possible solution. The *squares* depict metabolites, the *circles* represent reactions. The pathway is a feasible synthesis pathway from M1 to the product P

Figure 3 depicts an exemplary pathway to the target metabolite *P* illustrating a possible solution of the presented MILP.

The light yellow square **M1** is the starting metabolite of the linear path, whereas the dark orange square **P** is the target metabolite. The light blue squares are metabolites from the metabolite pool. The linear path highlighted in yellow is defined through constraints (1) to (6). One of the substrates for reaction **R3**, metabolite **M4**, is not available in the metabolite pool and thus must be supplied by other reactions. These supplying reactions are defined by constraints (7) to (12). In this example, reaction **R4** depicted by the white circle is added to the resulting path. The overall pathway is a synthesis pathway from M1 to the desired product P that is feasible within the given network.

### Filtering and ranking

We rank the pathway candidates generated by the MILP by different criteria in order to highlight the most meaningful candidates for the synthesis of the desired product. As a global optimization method, the MILP cannot take into account if the first reaction of a pathway candidate is feasible only with metabolites in the metabolite pool. We thus have to perform a filtering step before the ranking to eliminate those pathway candidates that do not comply with this requirement. The ranking criteria are listed in Table 1.

**Table 1:** Ranking criteria in the order they are applied to the pathway candidates

Position	Criterion	Comment
1	Number of active reactions	Shorter pathways are
		favourable
2	Candidate starts with basic metabolites only	’yes’ is preferred
3	Number of reactions without *Δ*_*r*_ *G*	As few as possible
4	$\sum (\Delta _{r}G + |\Delta _{r}G|)$	Preferably all *Δ*_*r*_ *G* are
		negative
5	$\sum \Delta _{r}G$	Negative is preferred
6	Number of heterologous enzymes	As few as possible
7	Number of cofactors	As few as possible

The first criterion is the number of active reactions in the pathway candidate. Shorter pathways favor a fast product formation, a reduced substrate demand and are generally easier to realize than a pathway with more reactions. The second ranking criterion prefers pathway candidates starting with basic metabolites only.

A further ranking criterion favors pathways for which there is thermodynamic information available. This is based on the notion that reactions without known or assessable *Δ*_*r*_ *G* are often poorly described. Another ranking criterion is the sum of the *Δ*_*r*_ *G*’s and the absolute value of those *Δ*_*r*_ *G*’s $\sum _{r}(\Delta _{r}G + |\Delta _{r}G|)$ for all reactions r in the linear path of the pathway candidate. Ideally this sum is 0, since then each reaction has a negative *Δ*_*r*_ *G*. Therefore, pathway candidates with positive *Δ*_*r*_ *G* of intermediate reactions are ranked down, as they would lead to kinetic traps. Furthermore, the pathway candidates are ranked by the overall thermodynamics of the linear path of the pathway candidate. Pathways with a negative overall *Δ*_*r*_ *G* are preferred over those with a positive overall *Δ*_*r*_ *G*.

The ranking also takes into account the number of enzymes that are native in a specified host organism. Pathways with less heterologous enzymes are preferred as they potentially require less genetic engineering work in the practical implementation.

The last ranking criterion counts the number of different cofactor species that are required by a pathway candidate. Cofactors are often expensive and require regeneration which can be difficult to implement. Thus, pathway candidates with less cofactors are preferred.

In addition to the output of the reactions of each pathway candidate and an overall balance of each reactant in a pathway, further information useful for their assessment is given. The thermodynamic profile allows for a quick visual assessment of each pathway.

An SBML [30] file containing all reactions on the pathway allows the visualization of the path and the active reactions with any tool capable of reading SBML (e.g. Cytoscape [31, 32]).

A list of possible side reactions for each pathway candidate in a given host organism can help to find pathways with a small number of side reactions or even identify those side reactions that can be deleted.

### Computational details

Our path-finding tool is implemented in MATLAB^Ⓒ^ R2015a (8.5.0) (MathWorks). As a MILP solver we used the IBM CPLEX Optimizer 12.5. All data from KEGG is obtained using the KEGG REST API. The eQuilibrator 2.0 source code was cloned from their GitHub repository [33].

All computations were carried out on a 64 bit, 3.4 Ghz Intel Core i7-2600 PC with 8 GB RAM.

## Results

**Fig. 4:**
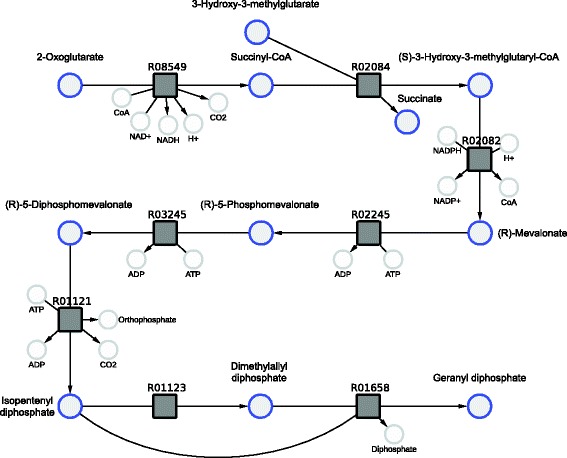
Pathway candidate 1. Synthesis of geranyl pyrophosphate via the mevalonate pathway

**Fig. 5:**
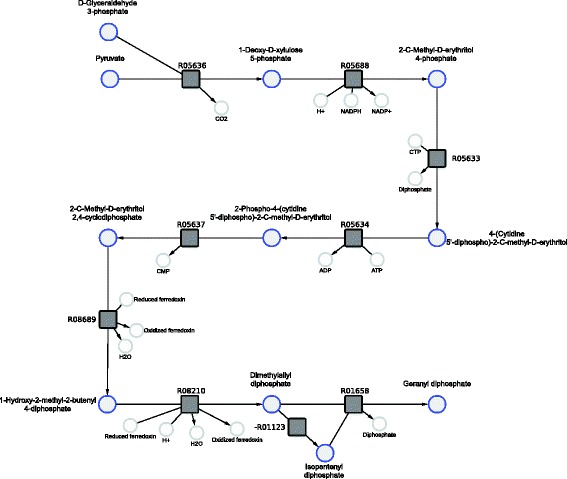
Pathway candidate 2. Synthesis of geranyl pyrophosphate via the non-mevalonate pathway

**Fig. 6:**
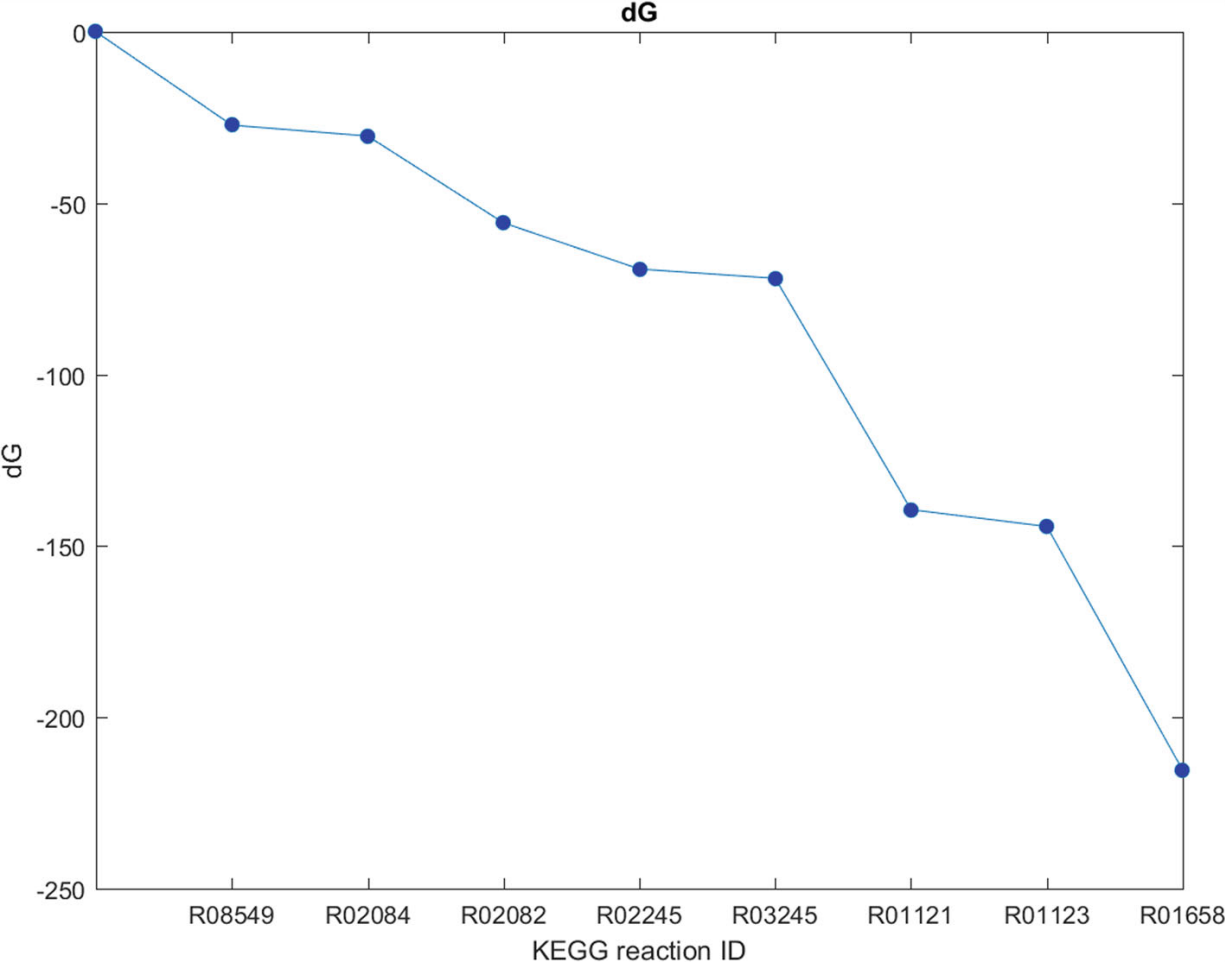
Thermodynamic profile for the mevalonate pathway

**Fig. 7:**
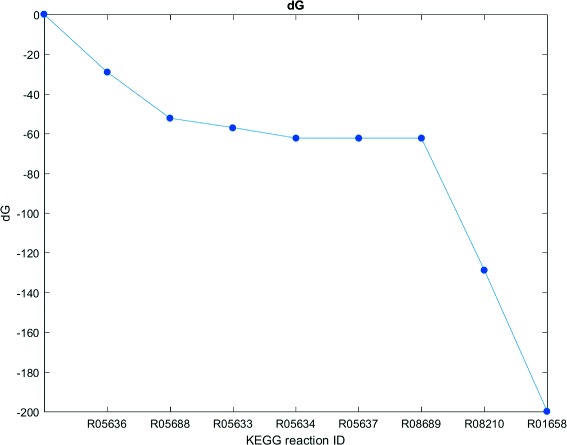
Thermodynamic profile for the non-mevalonate pathway

We use geranyl pyrophosphate (GPP) as a first example to illustrate features of our method. Geranyl pyrophosphate is part of the metabolism of most organisms and plays a key role in the terpenoid biosynthesis. Its precursors isopentenyl pyrophosphate (IPP) and dimethylallyl pyrophosphate (DMAPP) can be synthesized via two different pathways. The mevalonate pathway starting with acetyl-CoA is present in fungi, archaea and some bacteria. The non-mevalonate pathway (MEP/DOXP pathway) with pyruvate as a precursor exists in plants, eubacteria and protozoa [34]. From the computed pathways we chose interesting candidates depicted in Figs. 4 and 5. The pathway candidate in Fig. 4 corresponds to the lower mevalonate pathway. It starts with 2-oxoglutarate synthesizing IPP and DMAPP in seven consecutive reactions plus an additional reaction to GPP. The pathway candidate has 11 potential side reactions which are provided in more detail in the Additional file 2. These reactions can potentially be active in permeabilized cells or cell lysates but might be disrupted by corresponding gene deletions. If a synthetic mixture of enzymes of interest would be applied, these reactions would not be active at all. With the presented network we were also able to recover the non-mevalonate pathway shown in Fig. 5. The thermodynamic profiles for the linear path of these pathways are shown in Figs. 6 and 7. They indicate that the operation of these pathways is thermodynamically feasible with negative and constantly dropping *Δ*_*r*_ *G*. Our tool proposes 11 potential side reactions for the mevalonate pathway and 24 for the non-mevalonate pathway. They are provided in more detail in the Additional file 2. The candidate for the mevalonate pathway was chosen because of its favorable thermodynamic profile (Fig. 6) with a large drop of *Δ*_*r*_ *G* in the last two reactions. This final drop has the potential to lead to high conversion. Additionally, all substrates for the synthesis are readily available. However, the mevalonate pathway is not natively present in our chosen host *E. coli*. The second pathway candidate based on the non-mevalonate pathway displays an alternative method for the production of GPP, which is fully present in *E. coli*.

We chose amygdalin as a further example. In this case, we added sucrose as a potential starting and basis metabolite. Sucrose is excluded from the original set of starting metabolites because of its higher molecular mass but is much cheaper than *α*-D-glucose 6-phosphate. The generated pathways contain two interesting candidates with both four consecutive active reactions to amygdalin. The first candidate starts with sucrose and the second with *α*-D-glucose 6-phosphate. Both candidates require a uridyl moiety as substrate. Nevertheless, in the search carried out, UTP, UDP and UMP were considered cofactors to avoid unnecessary interconversion of nucleotides that would add numerous but not meaningful pathway candidates. And in both candidates, two of the reactions are catalyzed by heterologous enzymes. For the first pathway, four potential side reactions are proposed and five for the second. These pathway candidates highlight the impact of the list of potential starting metabolites on the results. While both pathways look promising, the first one starts with the cheap starting substrate sucrose and has a better thermodynamic profile. In an industrial environment it would be advisable to create a customized list of starting metabolites considering more criteria, e.g. of cost and availability.

Another example is pyrrolysine. The selected pathway candidate has four active reactions and starts with L-Lysine as substrate. Thermodynamic data for this pathway is not available in eQuilibrator. In *E. coli*, this pathway does not exist, but it is native in methanogenic archaea. The pathway requires ATP and NAD ^+^ /NADH as cofactors. It has nine potential side reactions.

As a last example, we chose (S)-2-phenyloxirane. The selected pathway candidate for (S)-2-phenyloxirane has four consecutive active reactions. It uses cinnamaldehyde as substrate and requires CoA, NADP ^+^ /NADPH and AxP as cofactors. The thermodynamic profile is not ideal with regard to the first and last reaction steps that both have a slightly positive *Δ*_*r*_ *G*. Potentially, the last step could be promoted by an efficient FADH_2_ regeneration or oxygen supply pushing the equilibrium to the product side. However, it remains questionable if FADH_2_ can be regenerated in permeabilized cells. Details to all examples shown are given in the respective sections of the Additional file 2. The Additional file 3 contains details on the computation times of all examples.

## Discussion

We presented a method for searching potential synthesis pathways for target metabolites without the specification of a fixed starting point. Due to the nature of the search algorithm, the resulting pathway candidates are unbiased by the user’s knowledge and expectation of the most suitable pathway. Our method leads to a large number of results in a broad solution space which may make it challenging to find the most appropriate candidate. Handling this amount of data requires a sophisticated tool of filtering, ranking and expert assessment together with additional features such as the quick evaluation of potential side reactions and thermodynamics. Altogether, our tool is highly customizable and offers flexible filtering and ranking options. All metabolite lists, especially the metabolite pool can be easily adapted to meet the needs of a specific project. This is especially useful in cases where the metabolite pool should be composed of chemicals of the laboratories’ inventory or of inexpensive chemicals. Analogously, all ranking or filtering criteria can be tailored to the focus of the study, such as reagent costs or a specific host organism.

Expert knowledge to assess the pathway candidates is still needed. However, the same applies to any pathway design method available to date. The resulting pathway candidates depend fully on the data used to set up the network. The sheer mass of reactions in KEGG makes errors hard to identify manually, and we did not carry out any data cleaning except the measures discussed in section Network reconstruction. Crude errors such as unbalanced or ill-formed reaction entries in KEGG were automatically identified and excluded from our network.

Thermodynamics of a pathway is complex. Most substances involved in a pathway are not present at the beginning but are rather formed as the synthesis proceeds. This is not taken into consideration. We fix the initial starting concentrations of all metabolites to 1 mM. However, these can be easily modified by adapting the respective values for the calculation of the *Δ*_*r*_ *G* in eQuilibrator. Note, that all *Δ*_*r*_ *G* are estimated using the component contribution method. They can however be replaced by experimental values, if available.

We do not consider enzyme concentrations or any kind of kinetic parameters such as enzyme turnover numbers or *K*_*m*_ values. While this would be a relevant addition, to our knowledge this information is not readily available on the scale needed for large networks. It could however be integrated for smaller networks, e. g. [35], particularly in the ranking procedure.

## Conclusions

The presented method provides a helpful computational tool for the directed design of biosynthetic production pathways and the planning of syntheses. The tool provides a very useful basis for the eventual selection of pathways to be implemented in the wet lab. Building on this, expert knowledge is required to tackle possible practical problems with the implementation of the most promising candidates. All features presented are autonomous. The generated thermodynamic profiles of pathways are invaluable for selecting the most promising pathway alternatives. Similarly, computing potential side reactions leads to important insights for all kinds of pathways.

In different use cases different ranking criteria may be considered important. The user of the tool can easily select or define own criteria for ranking results. For the synthesis with cell lysates or permeabilized cells, the consideration of heterologous enzymes and the choice of the most suitable host as well as potential side reactions are certainly very important.

## Endnotes

^1^ Discontinued since KEGG release 80.0, October 1, 2016

^2^ KEGG compound ID

## Additional files

Additional file 1 Lists of reactions and metabolites in the presented genome-scale metabolic network. The spreadsheets in the file provide all relevant data of our presented genome-scale metabolic network including all reactions, the subset of reactions present in *E. coli*, and all metabolite lists as defined in section Network details, as well as all arcs. (ODS 661 kb)

Additional file 2 Pathway candidates. The file presents the details of the presented pathway candidates, i.e. a list of all reactions involved, overall balances thermodynamic profiles and potential side reactions. (PDF 145 kb)

Additional file 3 Computation time of pathway candidates. The file presents details on the computation time of the different examples we presented. (PDF 338 kb)

## Abbreviations

B: Set of basis metabolites
E: Set of external metabolites
*E*_*m*_: Metabolite pool
GPP: Geranyl pyrophosphate
I: Set of internal metabolites
KEGG: Kyoto Encyclopedia of genes and genomes
M: Set of metabolites in the network
Max: Maximum non-negative integer flux value for all reactions R
MILP: Mixed-integer linear program
P: Product
PEP: phosphoenolpyruvate
R: Set of reactions in the network
S: Set of possible start metabolites
SBML: Systems biology markup language
T: Target metabolite
