# Supplementary material for: Network design and analysis for multi-enzyme biocatalysis
Source: BMC Bioinformatics. 2017 Aug 10;18:366. doi: 10.1186/s12859-017-1773-y (PMC5553788; doi:10.1186/s12859-017-1773-y)
Supplement: Supplementary file 2 — Pathway candidates. The file presents the details of the presented pathway candidates, i.e. a list of all reactions involved, overall balances thermodynamic profiles and potential side reactions. (PDF 145 kb) [file 12859_2017_1773_MOESM2_ESM.pdf]

Additional file 2: Pathway examples

Contents

1 geranyl pyrophosphate **2**
- 1.1 pathway candidate 1: mevalonate pathway 2
- 1.2 overall balance 4
- 1.3 side reactions 6
- 1.4 pathway candidate: non-mevalonate pathway 9
- 1.5 overall balance 11
- 1.6 side reactions 13

2 amygdalin **19**
- 2.1 pathway candidate: starting from sucrose 19
- 2.2 thermodynamic profile 21
- 2.3 overall balance 22
- 2.4 side reactions 24
- 2.5 pathway candidate: starting from α-D-Glucose 6-phosphate . . . 25
- 2.6 thermodynamic profile 27
- 2.7 overall balance 28
- 2.8 side reactions 30

3 pyrrolysine **31**
- 3.1 pathway candidate 31
- 3.2 thermodynamic profile 33
- 3.3 overall balance 34
- 3.4 side reactions 36

4 (S)-2-Phenyloxirane **39**
- 4.1 pathway candidate 39
- 4.2 thermodynamic profile 41
- 4.3 overall balance 42
- 4.4 side reactions 44

1 geranyl pyrophosphate

1.1 pathway candidate 1: mevalonate pathway

```
number of active reactions: 8
starts with basic: False
reactions w/o dG: 0
sum (dG + |dG|):0
dG: -2.154517e+02
number of heterologeous enzymes: 5
number of cofactors: 8

number of side reactions: 11

R08549 : 2-Oxoglutarate dehydrogenase complex    -2.723933
    e+01

        substrates:
                1       C00026  2-Oxoglutarate
                1       C00010  CoA
                1       C00003  NAD+

        products:
                1       C00091  Succinyl-CoA
                1       C00011  CO2
                1       C00004  NADH
                1       C00080  H+

R02084 : succinyl-CoA:3-hydroxy-3-methylglutarate CoA-
    transferase       -3.166626e+00

        substrates:
                1       C00091  Succinyl-CoA
                1       C03761  3-Hydroxy-3-
                    methylglutarate

        products:
                1       C00042  Succinate
                1       C00356  (S)-3-Hydroxy-3-
                    methylglutaryl-CoA

R02082 : (R)-Mevalonate:NADP+ oxidoreductase (CoA
    acylating)     -2.534910e+01

        substrates:
                1       C00356  (S)-3-Hydroxy-3-
```

```
              methylglutaryl-CoA
          2          C00005   NADPH
          2          C00080   H+

     products:
          1          C00418   (R)-Mevalonate
          1          C00010   CoA
          2          C00006   NADP+

R02245 : ATP:(R)-mevalonate 5-phosphotransferase
   -1.348146e+01

     substrates:
          1          C00002   ATP
          1          C00418   (R)-Mevalonate

     products:
          1          C00008   ADP
          1          C01107   (R)-5-Phosphomevalonate

R03245 : ATP:(R)-5-phosphomevalonate phosphotransferase
   -2.712405e+00

     substrates:
          1          C00002   ATP
          1          C01107   (R)-5-Phosphomevalonate

     products:
          1          C00008   ADP
          1          C01143   (R)-5-Diphosphomevalonate

R01121 : ATP:(R)-5-diphosphomevalonate carboxy-lyase (
   adding ATP          -6.741552e+01

     substrates:
          1          C00002   ATP
          1          C01143   (R)-5-Diphosphomevalonate

     products:
          1          C00008   ADP
          1          C00009   Orthophosphate
          1          C00129   Isopentenyl diphosphate
          1          C00011   CO2

R01123 : Isopentenyl-diphosphate delta3-delta2-isomerase
        -4.902559e+00
```

```
        substrates:
                1        C00129   Isopentenyl diphosphate

        products:
                1        C00235   Dimethylallyl diphosphate

R01658 : Dimethylallyl−diphosphate:isopentenyl−
    diphosphate dimethylallyltranstransferase
    −7.118467e+01

        substrates:
                1        C00235   Dimethylallyl diphosphate
                1        C00129   Isopentenyl diphosphate

        products:
                1        C00013   Diphosphate
                1        C00341   Geranyl diphosphate
```

1.2 overall balance

Table 1: Overall balance of the pathway candidate representing the mevalonate pathway

	R01121	R01123	R01658	R02082	R02084	R02245	R03245	R08549	overall
ATP	-1	0	0	0	0	-1	-1	0	-3
(R)-5-Diphosphomevalonate	-1	0	0	0	0	0	1	0	0
ADP	1	0	0	0	0	1	1	0	3
Orthophosphate	1	0	0	0	0	0	0	0	1
Isopentenyl diphosphate	1	-1	-1	0	0	0	0	0	-1
CO2	1	0	0	0	0	0	0	1	2
Dimethylallyl diphosphate	0	1	-1	0	0	0	0	0	0
Diphosphate	0	0	1	0	0	0	0	0	1
Geranyl diphosphate	0	0	1	0	0	0	0	0	1
(S)-3-Hydroxy-3-methylglutaryl-CoA	0	0	0	-1	1	0	0	0	0
NADPH	0	0	0	-2	0	0	0	0	-2
H+	0	0	0	-2	0	0	0	1	-1
(R)-Mevalonate	0	0	0	1	0	-1	0	0	0
CoA	0	0	0	1	0	0	0	-1	0
NADP+	0	0	0	2	0	0	0	0	2
Succinyl-CoA	0	0	0	0	-1	0	0	1	0
3-Hydroxy-3-methylglutarate	0	0	0	0	-1	0	0	0	-1
Succinate	0	0	0	0	1	0	0	0	1
(R)-5-Phosphomevalonate	0	0	0	0	0	1	-1	0	0
2-Oxoglutarate	0	0	0	0	0	0	0	-1	-1
NAD+	0	0	0	0	0	0	0	-1	-1
NADH	0	0	0	0	0	0	0	1	1

1.3 side reactions

```
R00089 : ATP diphosphate-lyase (cyclizing

        substrates:
                1       C00002  ATP

        products:
                1       C00575  3',5'-Cyclic AMP
                1       C00013  Diphosphate

R00104 : ATP:NAD+ 2'-phosphotransferase

        substrates:
                1       C00002  ATP
                1       C00003  NAD+

        products:
                1       C00008  ADP
                1       C00006  NADP+

R00112 : NADPH:NAD+ oxidoreductase

        substrates:
                1       C00005  NADPH
                1       C00003  NAD+

        products:
                1       C00006  NADP+
                1       C00004  NADH

R00405 : Succinate:CoA ligase (ADP-forming)

        substrates:
                1       C00002  ATP
                1       C00042  Succinate
                1       C00010  CoA

        products:
                1       C00008  ADP
                1       C00009  Orthophosphate
                1       C00091  Succinyl-CoA

R02003 : Geranyl-diphosphate:isopentenyl-diphosphate
    geranyltrans-transferase
```

```
        substrates:
                1         C00341   Geranyl diphosphate
                1         C00129   Isopentenyl diphosphate

        products:
                1         C00013   Diphosphate
                1         C00448   trans,trans-Farnesyl
                    diphosphate

-R00127 : -ATP:AMP phosphotransferase

        substrates:
                2         C00008   ADP

        products:
                1         C00002   ATP
                1         C00020   AMP

-R00130 : -ATP:dephospho-CoA 3'-phosphotransferase

        substrates:
                1         C00008   ADP
                1         C00010   CoA

        products:
                1         C00002   ATP
                1         C00882   Dephospho-CoA

-R00137 : -ATP:nicotinamide-nucleotide
    adenylyltransferase

        substrates:
                1         C00013   Diphosphate
                1         C00003   NAD+

        products:
                1         C00002   ATP
                1         C00455   Nicotinamide D-
                    ribonucleotide

-R00267 : -Isocitrate:NADP+ oxidoreductase (
    decarboxylating)

        substrates:
                1         C00026   2-Oxoglutarate
                1         C00011   CO2
```

1 C00005 NADPH
1 C00080 H+

products:
1 C00311 Isocitrate
1 C00006 NADP+

−R00519 : −formate:NAD+ oxidoreductase

substrates:
1 C00080 H+
1 C00011 CO2
1 C00004 NADH

products:
1 C00058 Formate
1 C00003 NAD+

−R00833 : −(R)−Methylmalonyl−CoA CoA−carbonylmutase

substrates:
1 C00091 Succinyl−CoA

products:
1 C01213 (R)−Methylmalonyl−CoA

1.4 pathway candidate: non-mevalonate pathway

```
number of active reactions: 9
starts with basic: False
reactions w/o dG: 2
sum (dG + |dG|):0
dG: −1.998150e+02
number of heterologeous enzymes: 0
number of cofactors: 3

number of side reactions: 24

R05636 : 1−Deoxy−D−xylulose−5−phosphate pyruvate−lyase (
    carboxylating)   −2.901798e+01

        substrates:
                1         C00022   Pyruvate
                1         C00118   D−Glyceraldehyde 3−
                    phosphate

        products:
                1         C11437   1−Deoxy−D−xylulose 5−
                    phosphate
                1         C00011   CO2

R05688 : 1−Deoxy−D−xylulose−5−phosphate isomeroreductase
        −2.321112e+01

        substrates:
                1         C11437   1−Deoxy−D−xylulose 5−
                    phosphate
                1         C00005   NADPH
                1         C00080   H+

        products:
                1         C11434   2−C−Methyl−D−erythritol
                    4−phosphate
                1         C00006   NADP+

R05633 : CTP: 2−C−Methyl−D−erythritol 4−phosphate
    cytidylyltransferase   −4.729713e+00

        substrates:
                1         C11434   2−C−Methyl−D−erythritol
                    4−phosphate
                1         C00063   CTP
```

```
        products:
                1         C11435  4-(Cytidine 5'-diphospho)
                    -2-C-methyl-D-erythritol
                1         C00013  Diphosphate

R05634 : ATP:4-(Cytidine 5'-diphospho)-2-C-methyl-D-
    erythritol 2-phosphotransferase       -5.273891e+00

        substrates:
                1         C11435  4-(Cytidine 5'-diphospho)
                    -2-C-methyl-D-erythritol
                1         C00002  ATP

        products:
                1         C11436  2-Phospho-4-(cytidine 5'-
                    diphospho)-2-C-methyl-D-erythritol
                1         C00008  ADP

R05637 : 2-Phospho-4-(cytidine 5'-diphospho)-2-C-methyl-D
    -erythritol CMP-lyase (cyclizing)       0

        substrates:
                1         C11436  2-Phospho-4-(cytidine 5'-
                    diphospho)-2-C-methyl-D-erythritol

        products:
                1         C11453  2-C-Methyl-D-erythritol
                    2,4-cyclodiphosphate
                1         C00055  CMP

R08689 : (E)-4-hydroxy-3-methylbut-2-en-1-yl-diphosphate:
    oxidized ferredoxin oxidoreductase (hydrating) 0

        substrates:
                1         C11453  2-C-Methyl-D-erythritol
                    2,4-cyclodiphosphate
                2         C00138  Reduced ferredoxin

        products:
                1         C11811  1-Hydroxy-2-methyl-2-
                    butenyl 4-diphosphate
                1         C00001  H2O
                2         C00139  Oxidized ferredoxin

R08210 : dimethylallyl diphosphate:ferredoxin
```

```
oxidoreductase      −6.639758e+01

    substrates:
        1       C11811  1−Hydroxy−2−methyl−2−
            butenyl 4−diphosphate
        2       C00138  Reduced ferredoxin
        2       C00080  H+

    products:
        1       C00235  Dimethylallyl diphosphate
        1       C00001  Oxidized ferredoxin
        2       C00139  H2O

R01658 : Dimethylallyl−diphosphate:isopentenyl−
    diphosphate dimethylallyltranstransferase
    −7.118467e+01

    substrates:
        1       C00235  Dimethylallyl diphosphate
        1       C00129  Isopentenyl diphosphate

    products:
        1       C00013  Diphosphate
        1       C00341  Geranyl diphosphate
___________
−R01123 : −Isopentenyl−diphosphate delta3−delta2−
    isomerase       0

    substrates:
        1       C00235  Dimethylallyl diphosphate

    products:
        1       C00129  Isopentenyl diphosphate
```

1.5 overall balance

Table 2: Overall balance of the pathway candidate representing the non-mevalonate pathway

	R01658	R05633	R05634	R05636	R05637	R05688	R08210	R08689	-R01123	overall
Dimethylallyl diphosphate	-1	0	0	0	0	0	1	0	-1	-1
Isopentenyl diphosphate	-1	0	0	0	0	0	0	0	1	0
Diphosphate	1	1	0	0	0	0	0	0	0	2
Geranyl diphosphate	1	0	0	0	0	0	0	0	0	1
2-C-Methyl-D-erythritol 4-phosphate	0	-1	0	0	0	1	0	0	0	0
CTP	0	-1	0	0	0	0	0	0	0	-1
4-(Cytidine 5-diphospho)-2-C-methyl-D-erythritol	0	1	-1	0	0	0	0	0	0	0
ATP	0	0	-1	0	0	0	0	0	0	-1
2-Phospho-4-(cytidine 5-diphospho)-2-C-methyl-D-erythritol	0	0	1	0	-1	0	0	0	0	0
ADP	0	0	1	0	0	0	0	0	0	1
Pyruvate	0	0	0	-1	0	0	0	0	0	-1
D-Glyceraldehyde 3-phosphate	0	0	0	-1	0	0	0	0	0	-1
1-Deoxy-D-xylulose 5-phosphate	0	0	0	1	0	-1	0	0	0	0
CO2	0	0	0	1	0	0	0	0	0	1
2-C-Methyl-D-erythritol 2,4-cyclodiphosphate	0	0	0	0	1	0	0	-1	0	0
CMP	0	0	0	0	1	0	0	0	0	1
NADPH	0	0	0	0	0	-1	0	0	0	-1
H+	0	0	0	0	0	-1	-2	0	0	-3
NADP+	0	0	0	0	0	1	0	0	0	1
1-Hydroxy-2-methyl-2-butenyl 4-diphosphate	0	0	0	0	0	0	-1	1	0	0
Reduced ferredoxin	0	0	0	0	0	0	-2	-2	0	-4
Oxidized ferredoxin	0	0	0	0	0	0	2	2	0	4
H2O	0	0	0	0	0	0	1	1	0	2

1.6 side reactions

```
R00004 : diphosphate phosphohydrolase

        substrates:
                1        C00013  Diphosphate
                1        C00001  H2O

        products:
                2        C00009  Orthophosphate

R00086 : ATP phosphohydrolase

        substrates:
                1        C00002  ATP
                1        C00001  H2O

        products:
                1        C00008  ADP
                1        C00009  Orthophosphate

R00087 : ATP diphosphohydrolase (diphosphate-forming)

        substrates:
                1        C00002  ATP
                1        C00001  H2O

        products:
                1        C00020  AMP
                1        C00013  Diphosphate

R00089 : ATP diphosphate-lyase (cyclizing

        substrates:
                1        C00002  ATP

        products:
                1        C00575  3,5-Cyclic AMP
                1        C00013  Diphosphate

R00199 : ATP:pyruvate,water phosphotransferase

        substrates:
                1        C00002  ATP
                1        C00022  Pyruvate
                1        C00001  H2O
```

```
products:
        1       C00020  AMP
        1       C00074  Phosphoenolpyruvate
        1       C00009  Orthophosphate

R00200 : ATP:pyruvate 2-O-phosphotransferase

    substrates:
        1       C00002  ATP
        1       C00022  Pyruvate

    products:
        1       C00008  ADP
        1       C00074  Phosphoenolpyruvate

R00511 : cytidine-5-monophosphate phosphohydrolase

    substrates:
        1       C00055  CMP
        1       C00001  H2O

    products:
        1       C00475  Cytidine
        1       C00009  Orthophosphate

R00512 : ATP:CMP phosphotransferase

    substrates:
        1       C00002  ATP
        1       C00055  CMP

    products:
        1       C00008  ADP
        1       C00112  CDP

R00515 : CTP diphosphohydrolase (diphosphate-forming)

    substrates:
        1       C00063  CTP
        1       C00001  H2O

    products:
        1       C00055  CMP
        1       C00013  Diphosphate
```

R00568 : CTP aminohydrolase

substrates:

1	C00063	CTP
1	C00001	H2O

products:

1	C00075	UTP
1	C00014	Ammonia

R00572 : CTP:pyruvate 2–O–phosphotransferase

substrates:

1	C00063	CTP
1	C00022	Pyruvate

products:

1	C00112	CDP
1	C00074	Phosphoenolpyruvate

R01015 : D–glyceraldehyde–3–phosphate aldose–ketose–isomerase

substrates:

1	C00118	D–Glyceraldehyde 3–phosphate

products:

1	C00111	Glycerone phosphate

R01123 : Isopentenyl–diphosphate delta3–delta2–isomerase

substrates:

1	C00129	Isopentenyl diphosphate

products:

1	C00235	Dimethylallyl diphosphate

R01195 : Ferredoxin:NADP+ oxidoreductase

substrates:

1	C00006	Reduced ferredoxin
1	C00080	NADP+
2	C00138	H+

products:

```
                1         C00005   Oxidized  ferredoxin
                2         C00139   NADPH

R02003  :  Geranyl−diphosphate:isopentenyl−diphosphate
   geranyltrans−transferase

        substrates:
                1         C00341   Geranyl  diphosphate
                1         C00129   Isopentenyl  diphosphate

        products:
                1         C00013   Diphosphate
                1         C00448   trans,trans−Farnesyl
                    diphosphate

R05884  :  isopentenyl−diphosphate:ferredoxin
   oxidoreductase

        substrates:
                1         C11811   1−Hydroxy−2−methyl−2−
                    butenyl 4−diphosphate
                2         C00138   Reduced  ferredoxin
                2         C00080   H+

        products:
                1         C00129   Isopentenyl  diphosphate
                1         C00001   Oxidized  ferredoxin
                2         C00139   H2O

−R00104  :  −ATP:NAD+ 2−phosphotransferase

        substrates:
                1         C00008   ADP
                1         C00006   NADP+

        products:
                1         C00002   ATP
                1         C00003   NAD+

−R00127  :  −ATP:AMP  phosphotransferase

        substrates:
                2         C00008   ADP

        products:
                1         C00002   ATP
```

```
        1         C00020   AMP

−R00132 : −carbonate hydro−lyase (carbon−dioxide−forming)

    substrates:
        1         C00011   CO2
        1         C00001   H2O

    products:
        1         C01353   Carbonic acid

−R00216 : −(S)−Malate:NADP+ oxidoreductase(oxaloacetate−
  decarboxylating)

    substrates:
        1         C00022   Pyruvate
        1         C00011   CO2
        1         C00005   NADPH
        1         C00080   H+

    products:
        1         C00149   (S)−Malate
        1         C00006   NADP+

−R00513 : −ATP:cytidine 5−phosphotransferase

    substrates:
        1         C00008   ADP
        1         C00055   CMP

    products:
        1         C00002   ATP
        1         C00475   Cytidine

−R01064 : −2−dehydro−3−deoxy−6−phospho−D−galactonate D−
  glyceraldehyde−3−phospho−lyase (pyruvate−forming)

    substrates:
        1         C00022   Pyruvate
        1         C00118   D−Glyceraldehyde 3−
          phosphate

    products:
        1         C01286   2−Dehydro−3−deoxy−6−
          phospho−D−galactonate
```

−R05605 : −2−dehydro−3−deoxy−6−phospho−D−gluconate D−glyceraldehyde−3−phosphate−lyase (pyruvate−forming)

substrates:

1	C00118	D−Glyceraldehyde 3−phosphate
1	C00022	Pyruvate

products:

1	C04442	2−Dehydro−3−deoxy−6−phospho−D−gluconate

−R10092 : −carbonate hydro−lyase (carbon−dioxide−forming)

substrates:

1	C00011	CO2
1	C00001	H2O

products:

1	C00288	HCO3−
1	C00080	H+

2 amygdalin

2.1 pathway candidate: starting from sucrose

```
number of active reactions: 4
starts with basic: True
reactions w/o dG: 0
sum (dG + |dG|):9.763018e-02
dG: -4.130443e+01
number of heterologeous enzymes: 2
number of cofactors: 1
number of side reactions: 4

R00803 : sucrose:phosphate alpha-D-glucosyltransferase
    4.881509e-02

         substrates:
                  1        C00089   Sucrose
                  1        C00009   Orthophosphate

         products:
                  1        C00095   D-Fructose
                  1        C00103   D-Glucose 1-phosphate

R00289 : UTP:alpha-D-glucose-1-phosphate
    uridylyltransferase      -8.295754e+00

         substrates:
                  1        C00075   UTP
                  1        C00103   D-Glucose 1-phosphate

         products:
                  1        C00013   Diphosphate
                  1        C00029   UDP-glucose

R10638 : UDP-D-glucose:(R)-mandelonitrile beta-D-
    glucosyltransferase      -1.412458e+01

         substrates:
                  1        C00561   Mandelonitrile
                  1        C00029   UDP-glucose

         products:
                  1        C00844   Prunasin
                  1        C00015   UDP
```

R10639 : −1.893291e+01

substrates:

1	C00844	Prunasin
1	C00029	UDP−glucose

products:

1	C08325	Amygdalin
1	C00015	UDP

2.2 thermodynamic profile

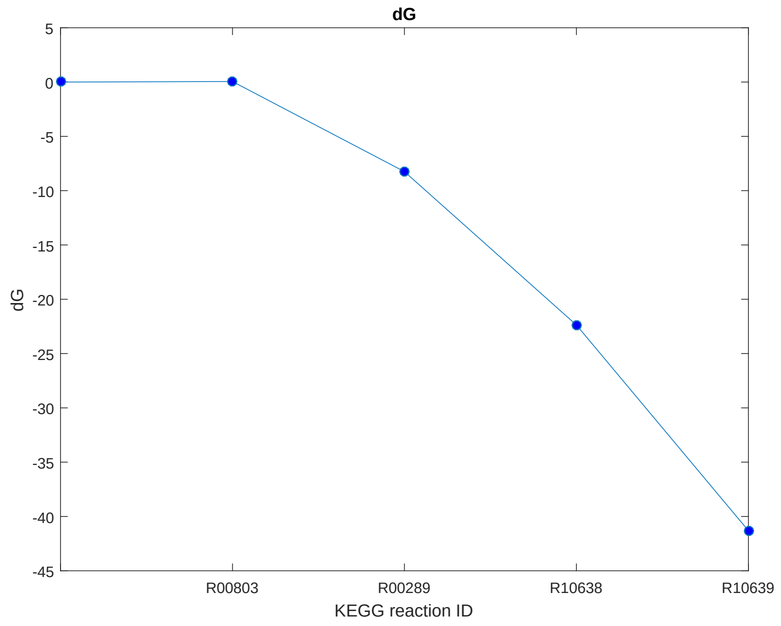

Figure 1: Thermodynamic profile for the pathway candidate for the synthesis of amygdalin from sucrose.

2.3 overall balance

```
reaction R00289

substrates:
        1 C00013
        1 C00029

products:
        1 C00075
        1 C00103

reaction R00803

substrates:
        1 C00095
        1 C00103

products:
        1 C00089
        1 C00009

reaction R10638

substrates:
        1 C00844
        1 C00015

products:
        1 C00561
        1 C00029

reaction R10639

substrates:
        1 C08325
        1 C00015

products:
        1 C00844
        1 C00029

overall:
```

C00009 1
C00013 −1
C00015 −2

```
C00029   1
C00075   1
C00089   1
C00095   −1
C00103   0
C00561   1
C00844   0
C08325   −1

not balanced:
C00009   1
C00013   −1
C00015   −2
C00029   1
C00075   1
C00089   1
C00095   −1
C00561   1
C08325   −1
```

2.4 side reactions

```
R00291 : UDP–glucose 4–epimerase

        substrates:
                1       C00029  UDP–glucose

        products:
                1       C00052  UDP–alpha–D–galactose

R00959 : alpha–D–Glucose 1–phosphate 1,6–phosphomutase

        substrates:
                1       C00103  D–Glucose 1–phosphate

        products:
                1       C00668  alpha–D–Glucose 6–phosphate

R08639 : alpha–D–glucose 1,6–phosphomutase

        substrates:
                1       C00103  D–Glucose 1–phosphate

        products:
                1       C00092  D–Glucose 6–phosphate

–R00878 : –alpha–D–Glucose aldose–ketose–isomerase

        substrates:
                1       C00095  D–Fructose

        products:
                1       C00267  alpha–D–Glucose
```

2.5 pathway candidate: starting from α-D-Glucose 6-phosphate

```
number of active reactions: 4
starts with basic: False
reactions w/o dG: 0
sum (dG + |dG|):2.070859e+00
dG: -3.202206e+01
number of heterologeous enzymes: 2
number of cofactors: 1
number of side reactions: 5

-R00959 : -alpha-D-Glucose 1-phosphate 1,6-phosphomutase
        4.881509e-02

    substrates:
            1       C00668  alpha-D-Glucose 6-
        phosphate

    products:
            1       C00103  D-Glucose 1-phosphate

R00289 : UTP:alpha-D-glucose-1-phosphate
    uridylyltransferase     -1.412458e+01

    substrates:
            1       C00075  UTP
            1       C00103  D-Glucose 1-phosphate

    products:
            1       C00013  Diphosphate
            1       C00029  UDP-glucose

R10638 : UDP-D-glucose:(R)-mandelonitrile beta-D-
    glucosyltransferase     -1.893291e+01

    substrates:
            1       C00561  Mandelonitrile
            1       C00029  UDP-glucose

    products:
            1       C00844  Prunasin
            1       C00015  UDP

R10639 :        9.866146e-01

    substrates:
```

```
            1           C00844   Prunasin
            1           C00029   UDP-glucose

products:
            1           C08325   Amygdalin
            1           C00015   UDP
```

2.6 thermodynamic profile

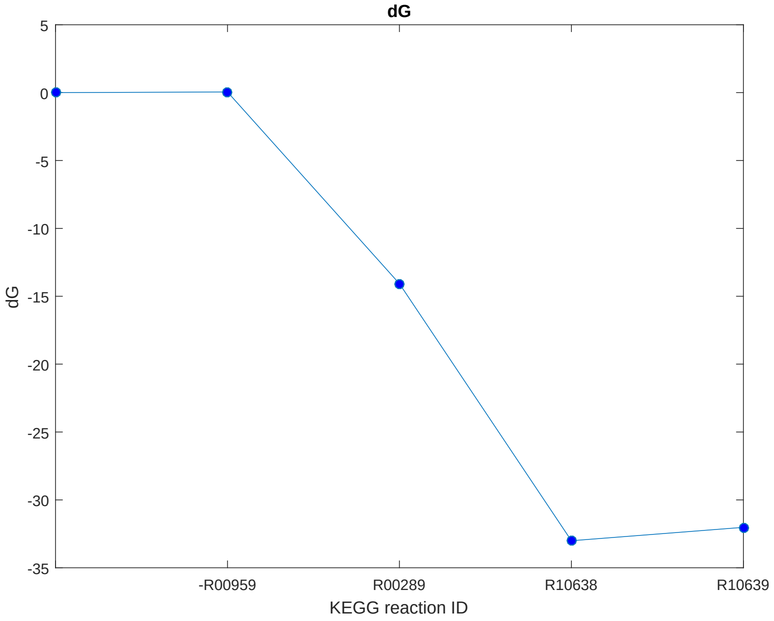

Figure 2: Thermodynamic profile for the pathway candidate for the synthesis of amygdalin from α-D-Glucose 6-phosphate.

2.7 overall balance

```
reaction R00289

substrates:
        1 C00013
        1 C00029

products:
        1 C00075
        1 C00103

reaction R10638

substrates:
        1 C00844
        1 C00015

products:
        1 C00561
        1 C00029

reaction R10639

substrates:
        1 C08325
        1 C00015

products:
        1 C00844
        1 C00029

reaction R00959

substrates:
        1 C00668

products:
        1 C00103

overall:
C00013  −1
C00015  −2
C00029  1
C00075  1
C00103  2
```

```
C00561  1
C00668  −1
C00844  0
C08325  −1

not balanced:
C00013  −1
C00015  −2
C00029  1
C00075  1
C00103  2
C00561  1
C00668  −1
C08325  −1
```

2.8 side reactions

```
R00291 : UDP-glucose 4-epimerase

        substrates:
                1       C00029  UDP-glucose

        products:
                1       C00052  UDP-alpha-D-galactose

R00959 : alpha-D-Glucose 1-phosphate 1,6-phosphomutase

        substrates:
                1       C00103  D-Glucose 1-phosphate

        products:
                1       C00668  alpha-D-Glucose 6-phosphate

R02737 : UDPglucose:D-glucose-6-phosphate 1-alpha-D-glucosyltransferase

        substrates:
                1       C00029  UDP-glucose
                1       C00668  alpha-D-Glucose 6-phosphate

        products:
                1       C00015  UDP
                1       C00689  alpha,alpha'-Trehalose 6-phosphate

R02740 : alpha-D-Glucose 6-phosphate ketol-isomerase

        substrates:
                1       C00668  alpha-D-Glucose 6-phosphate

        products:
                1       C05345  beta-D-Fructose 6-phosphate

R08639 : alpha-D-glucose 1,6-phosphomutase

        substrates:
                1       C00103  D-Glucose 1-phosphate

        products:
                1       C00092  D-Glucose 6-phosphate
```

3 pyrrolysine

3.1 pathway candidate

```
number of active reactions: 4
starts with basic: True
reactions w/o dG: 4
sum (dG + |dG|):0
dG: 0
number of heterologeous enzymes: 4
number of cofactors: 2
number of side reactions: 9

R10010 : L-lysine carboxy-aminomethylmutase      0

        substrates:
                1       C00047  L-Lysine

        products:
                1       C20277  (2R,3R)-3-Methylornithine

R10011 :        0

        substrates:
                1       C00047  L-Lysine
                1       C20277  (2R,3R)-3-Methylornithine
                1       C00002  ATP

        products:
                1       C20278  (2R,3R)-3-
                    Methylornithinyl-N6-lysine
                1       C07305  Products of ATP breakdown

R10012 :        0

        substrates:
                1       C20278  (2R,3R)-3-
                    Methylornithinyl-N6-lysine
                1       C00003  NAD+
                1       C00001  H2O

        products:
                1       C20279  (2R,3R)-3-Methylglutamyl
                    -5-semialdehyde-N6-lysine
                1       C00014  Ammonia
                1       C00004  NADH
```

R10013 : 0

substrates:

1 C20279 (2R,3R)–3–Methylglutamyl –5–semialdehyde–N6–lysine

products:

1 C16138 L–Pyrrolysine
1 C00001 H2O

3.2 overall balance

```
reaction R10010

substrates:
        1 C20277

products:
        1 C00047

reaction R10011

substrates:
        1 C20278
        1 C07305

products:
        1 C00047
        1 C20277
        1 C00002

reaction R10012

substrates:
        1 C20279
        1 C00014
        1 C00004

products:
        1 C20278
        1 C00003
        1 C00001

reaction R10013

substrates:
        1 C16138
        1 C00001

products:
        1 C20279

overall:
```

C00001 0
C00002 1

```
C00003   1
C00004   −1
C00014   −1
C00047   2
C07305   −1
C16138   −1
C20277   0
C20278   0
C20279   0

not balanced:
C00002   1
C00003   1
C00004   −1
C00014   −1
C00047   2
C07305   −1
C16138   −1
```

3.3 side reactions

```
R00086 : ATP phosphohydrolase

        substrates:
                1       C00002  ATP
                1       C00001  H2O

        products:
                1       C00008  ADP
                1       C00009  Orthophosphate

R00087 : ATP diphosphohydrolase (diphosphate-forming)

        substrates:
                1       C00002  ATP
                1       C00001  H2O

        products:
                1       C00020  AMP
                1       C00013  Diphosphate

R00089 : ATP diphosphate-lyase (cyclizing

        substrates:
                1       C00002  ATP

        products:
                1       C00575  3',5'-Cyclic AMP
                1       C00013  Diphosphate

R00103 : NAD+ phosphohydrolase

        substrates:
                1       C00003  NAD+
                1       C00001  H2O

        products:
                1       C00020  AMP
                1       C00455  Nicotinamide D-ribonucleotide

R00104 : ATP:NAD+ 2'-phosphotransferase

        substrates:
                1       C00002  ATP
                1       C00003  NAD+
```

```
        products:
                1          C00008   ADP
                1          C00006   NADP+

R00143 : ammonia:NAD+ oxidoreductase

        substrates:
                1          C00014   Ammonia
                1          C00003   NAD+
                1          C00001   H2O

        products:
                1          C00192   Hydroxylamine
                1          C00004   NADH
                1          C00080   H+

R00462 : L-lysine carboxy-lyase (cadaverine-forming)

        substrates:
                1          C00047   L-Lysine

        products:
                1          C01672   Cadaverine
                1          C00011   CO2

R00787 : ammonia:NAD+ oxidoreductase

        substrates:
                1          C00014   Ammonia
                2          C00001   NAD+
                3          C00003   H2O

        products:
                1          C00088   Nitrite
                3          C00004   NADH
                3          C00080   H+

R11104 : NADH phosphohydrolase

        substrates:
                1          C00004   NADH
                1          C00001   H2O

        products:
                1          C00020   AMP
```

1 C21113 NMNH

4 (S)-2-phenyloxirane

4.1 pathway candidate

```
number of active reactions: 4
starts with basic: False
reactions w/o dG: 1
sum (dG + |dG|):2.081496e+01
dG: −1.728943e+01
number of heterologeous enzymes: 4
number of cofactors: 3
number of side reactions: 11

R02506 : cinnamaldehyde:NADP+ oxidoreductase (CoA−
    cinnamoylating)        6.126122e+00

        substrates:
                1          C00903   Cinnamaldehyde
                1          C00010   CoA
                1          C00006   NADP+

        products:
                1          C00540   Cinnamoyl−CoA
                1          C00005   NADPH
                1          C00080   H+

−R02255 : −trans−Cinnamate:CoA ligase (AMP−forming)        0

        substrates:
                1          C00020   AMP
                1          C00013   Diphosphate
                1          C00540   Cinnamoyl−CoA

        products:
                1          C00002   ATP
                1          C00423   trans−Cinnamate
                1          C00010   CoA

R11070 : trans−cinnamate carboxy−lyase   −2.769690e+01

        substrates:
                1          C00423   trans−Cinnamate

        products:
                1          C07083   Styrene
                1          C00011   CO2
```

R05488 : styrene,FADH2:oxygen oxidoreductase 4.281356e+00

substrates:

1	C07083	Styrene
1	C01352	FADH2
1	C00007	Oxygen

products:

1	C20782	(S)−2−Phenyloxirane
1	C00016	FAD
1	C00001	H2O

4.2 thermodynamic profile

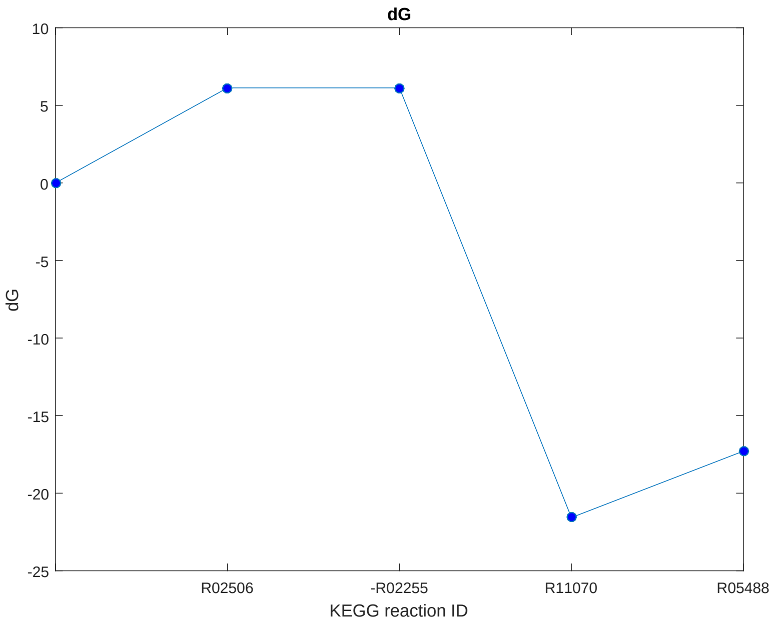

Figure 3: Thermodynamic profile for a pathway candidate for the synthesis of (S)-2-Phenyloxirane starting from cinnamaldehyde.

4.3 overall balance

```
reaction  R02506

substrates:
        1  C00540
        1  C00005
        1  C00080

products:
        1  C00903
        1  C00010
        1  C00006

reaction  R05488

substrates:
        1  C20782
        1  C00016
        1  C00001

products:
        1  C07083
        1  C01352
        1  C00007

reaction  R11070

substrates:
        1  C07083
        1  C00011

products:
        1  C00423

reaction  R02255

substrates:
        1  C00020
        1  C00013
        1  C00540

products:
        1  C00002
        1  C00423
        1  C00010
```

```
overall:
C00001  −1
C00002  1
C00005  −1
C00006  1
C00007  1
C00010  2
C00011  −1
C00013  −1
C00016  −1
C00020  −1
C00080  −1
C00423  2
C00540  −2
C00903  1
C01352  1
C07083  0
C20782  −1

not balanced:
C00001  −1
C00002  1
C00005  −1
C00006  1
C00007  1
C00010  2
C00011  −1
C00013  −1
C00016  −1
C00020  −1
C00080  −1
C00423  2
C00540  −2
C00903  1
C01352  1
C20782  −1
```

4.4 side reactions

```
R00004 : diphosphate phosphohydrolase

        substrates:
                1          C00013   Diphosphate
                1          C00001   H2O

        products:
                2          C00009   Orthophosphate

R00086 : ATP phosphohydrolase

        substrates:
                1          C00002   ATP
                1          C00001   H2O

        products:
                1          C00008   ADP
                1          C00009   Orthophosphate

R00087 : ATP diphosphohydrolase (diphosphate-forming)

        substrates:
                1          C00002   ATP
                1          C00001   H2O

        products:
                1          C00020   AMP
                1          C00013   Diphosphate

R00089 : ATP diphosphate-lyase (cyclizing

        substrates:
                1          C00002   ATP

        products:
                1          C00575   3',5'-Cyclic AMP
                1          C00013   Diphosphate

R00127 : ATP:AMP phosphotransferase

        substrates:
                1          C00002   ATP
                1          C00020   AMP
```

```
        products:
                2        C00008  ADP

R00182 : AMP phosphoribohydrolase

        substrates:
                1        C00020  AMP
                1        C00001  H2O

        products:
                1        C00147  Adenine
                1        C00117  D-Ribose 5-phosphate

R00183 : adenosine 5'-monophosphate phosphohydrolase

        substrates:
                1        C00020  AMP
                1        C00001  H2O

        products:
                1        C00212  Adenosine
                1        C00009  Orthophosphate

R00190 : AMP:diphosphate phospho-D-ribosyltransferase

        substrates:
                1        C00020  AMP
                1        C00013  Diphosphate

        products:
                1        C00147  Adenine
                1        C00119  5-Phospho-alpha-D-ribose 1-diphosphate

-R00132 : -carbonate hydro-lyase (carbon-dioxide-forming)

        substrates:
                1        C00011  CO2
                1        C00001  H2O

        products:
                1        C01353  Carbonic acid

-R00161 : -ATP:FMN adenylyltransferase

        substrates:
                1        C00013  Diphosphate
```

```
            1           C00016   FAD

        products:
            1           C00002   ATP
            1           C00061   FMN

−R10092 : −carbonate hydro−lyase (carbon−dioxide−forming)

        substrates:
            1           C00011   CO2
            1           C00001   H2O

        products:
            1           C00288   HCO3−
            1           C00080   H+
```
