# Supplementary material for: Network design and analysis for multi-enzyme biocatalysis
Source: BMC Bioinformatics. 2017 Aug 10;18:366. doi: 10.1186/s12859-017-1773-y (PMC5553788; doi:10.1186/s12859-017-1773-y)
Supplement: Supplementary file 3 — Computation time of pathway candidates. The file presents details on the computation time of the different examples we presented. (PDF 338 kb) [file 12859_2017_1773_MOESM3_ESM.pdf]

Additional file 3: Computation times

Contents

1 geranyl pyrophosphate **2**

2 amygdalin **3**

3 pyrolysine **4**

4 (S)-2-phenyloxirane **5**

5 Remarks **6**

1 geranyl pyrophosphate

We generated 1645 pathway candidates.

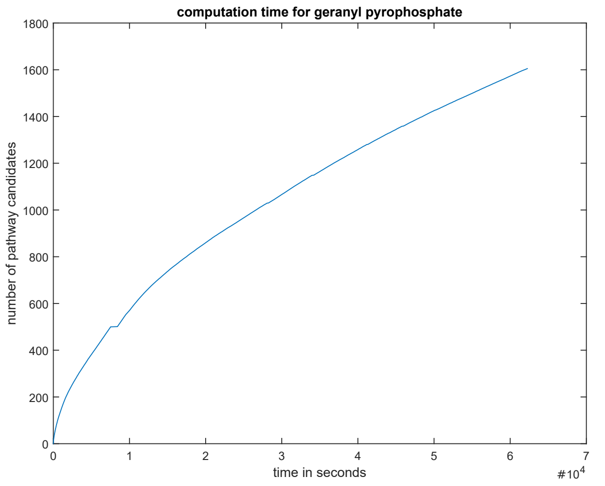

Figure 1: Number of pathway candidates over time for geranyl pyrophosphate.

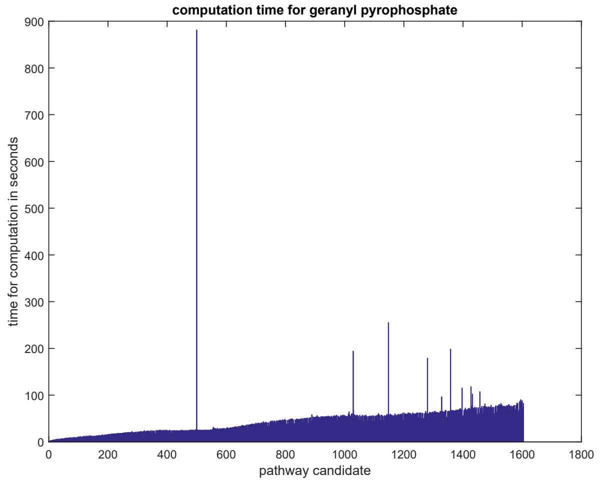

Figure 2: Computation time for individual pathway candidates for geranyl pyrophosphate.

2 amygdalin

We generated 100 pathway candidates.

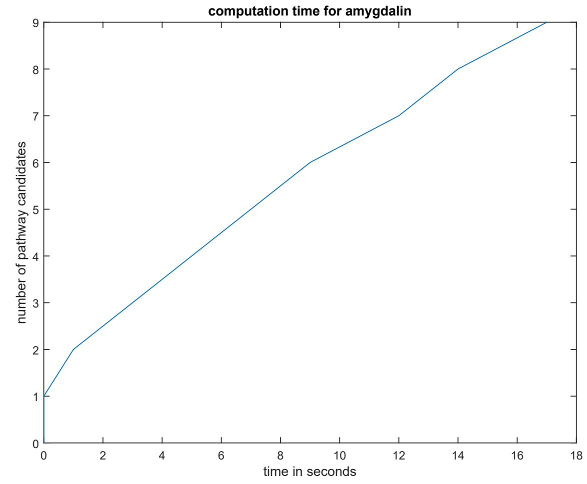

Figure 3: Computation time for individual pathway candidates for amygdalin.

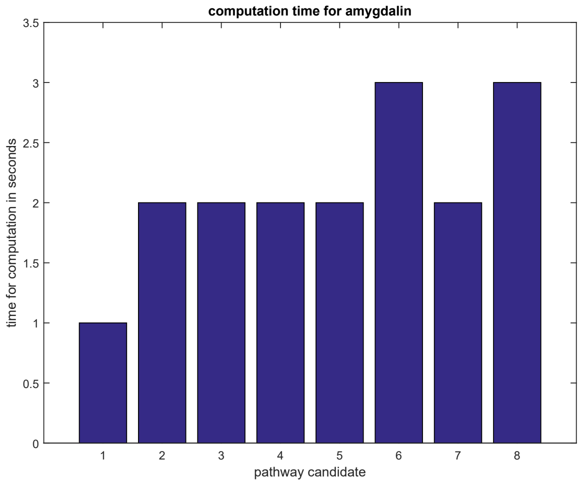

Figure 4: Number of pathway candidates over time for amygdalin.

3 pyrolysine

5 pathway candidates were generated.

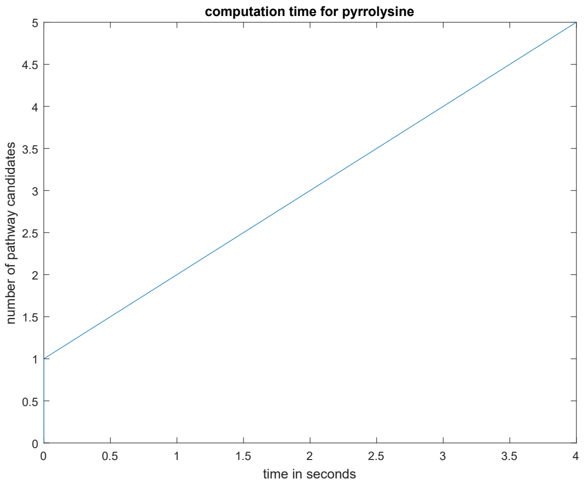

Figure 5: Number of pathway candidates over time for pyrrolysine.

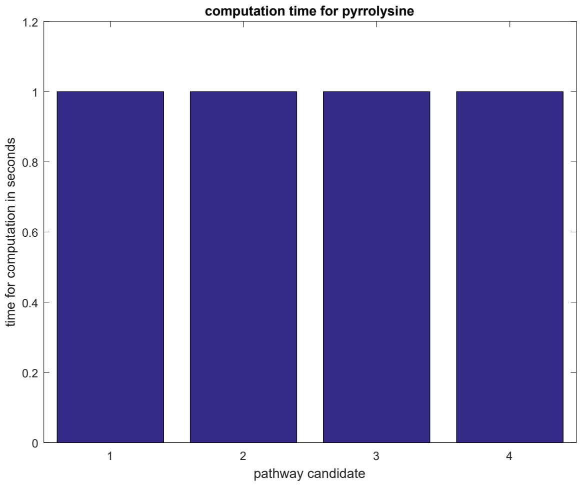

Figure 6: Computation time for individual pathway candidates for pyrrolysine.

4 (S)-2-phenyloxirane

11 pathway candidates were generated.

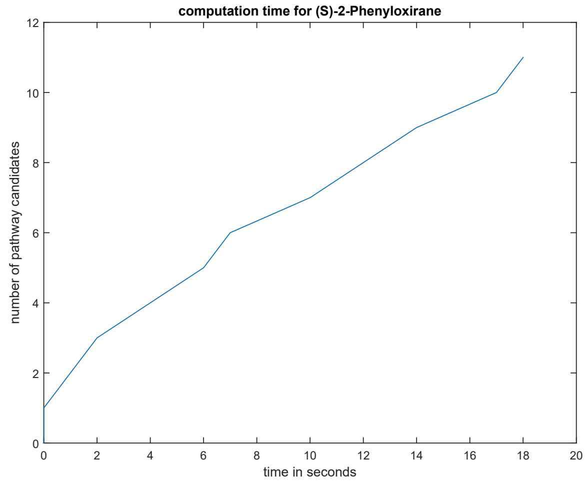

Figure 7: Number of pathway candidates over time for (S)-2-Phenyloxirane.

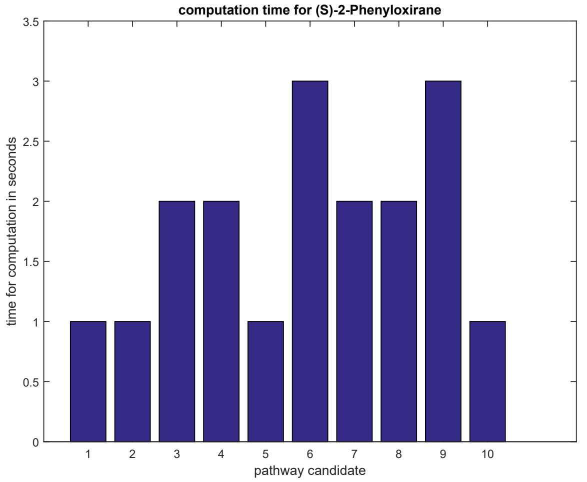

Figure 8: Computation time for individual pathway candidates for (S)-2-Phenyloxirane.

5 Remarks

We showed the number of pathway candidates over time empirically for the example pathway searches we presented in the paper. For a typical pathway search, the computation time was in the range of minutes for the first 100 pathway candidates. For all examples, the trend shows that the solver takes more time with each solution, due to the fact that for each additional solution, the number of constraint and variables in the MILP grows.
